# Avoidable injuries after intervention for abdominal aortic aneurysm: An analysis of negligence claims over 15 years in Sweden

**DOI:** 10.48101/ujms.v130.12171

**Published:** 2025-07-22

**Authors:** David Bergqvist, Pelle Gustafson, Larsolof Hafström

**Affiliations:** a Department of Surgical Sciences, Section of Surgery, Academic Hospital, Uppsala University, Uppsala, Sweden; b Swedish National Patient Insurance Company, Stockholm, Sweden; c Department of Surgery, Sahlgrenska Hospital, Gothenburg, Sweden

**Keywords:** Aortic aneurysm, surgery, endovascular treatment, complications, adverse events, negligence

## Abstract

**Background:**

Patients, who are subjected to a patient's injury, are legally allowed a compensation for their suffering.

**Aim:**

The negligence claims after surgical treatment of abdominal aortic aneurysms (AAA) registered in the National Swedish patient insurance company (Landstingens Ömsesidiga Försäkringsbolag [LÖF]) between 2006 and 2020 were analyzed. More than 95% of negligence claims are covered by LÖF. Special emphasis on avoidable or unavoidable injuries was made.

**Material:**

In 15 years 17,000 abdominal aortic interventions were recorded in the Swedish vascular register (SWEDVASC), where vascular interventions in the whole country of Sweden are registered. A total of 151 negligence claims (0.9%) were reported to the insurance company. Available clinical information in the company’s file of the claims was analyzed. The SWEDVASC data on AAA treatment were accessible.

**Results:**

The number of Endovascular repair (EVAR) increased significantly, but the total number of interventions decreased. There were less claims after EVAR (0.7%) compared to open surgery (1.1%). There was an increase in avoidable injuries that were economically compensated (*p* = 0.02). Spinal cord ischemia and intestinal ischemia were dominating causes for claims

**Conclusion:**

The increase in the number of *avoidable* injuries should have an impact on how to train and support colleagues under education and efforts to diminish the injuries are essential. To develop methods to diminish the risk for *non-avoidable* complications is important.

## Introduction

Open surgical treatment of abdominal aortic aneurysm (AAA) has been used since the introduction in the mid-1950s, Endovascular repair (EVAR) has been increasingly used since the mid-1990s. Both methods are accepted as standard procedures with the main purpose to prevent a potentially lethal aneurysmal rupture. Both strategies go with operative risks of varying severity, avoidable and non-avoidable (1).

During the last three decades, there have been some dramatic changes regarding AAA and these could potentially influence the negligence claims raised by patients having undergone treatment for AAA. These changes are the increasing use of EVAR (endovascular aneurysm repair) instead of open surgery not only in both elective cases but also in ruptured ones, the increasing use of ultrasonographic population screening programs and the decreasing prevalence of the disease (2).

Since 1994, a vascular treatment registry (SWEDVASC) covering the whole of Sweden has been used and more than 95% of all AAA interventions are registered (3). This registry is very accurate concerning the number of treatments, type of intervention and various complications (3).

Patients treated within the Swedish medical service system can claim negligence injuries to the malpractice insurance review board and request financial compensation for their suffering and financial losses due to an avoidable injury. In previous reports, shorter periods of time have been covered and have been less detailed (4, 5). There was an increase in negligence claims but not in compensated ones. The increased use of endovascular procedures was not influencing the pattern of compensated negligence injuries (5).

The aim of this study was to analyze claims due to clinical negligence after invasive intervention for AAA over a 15-years period and to analyze whether there has been a change in claims pattern during this period. Special emphasis was directed concerning the judge verdict of avoidable injuries, which were economically compensated, and if there was any difference between open and endovascular strategies in these aspects.

## Methods and materials

A retrospective observational study was performed. The files of negligence claims to the National Swedish Patient Insurance Company (LÖF, www.lof.se/english) regarding interventions with the diagnosis AAA in the years 2006–2020 were analyzed in three 5-year periods. Claims related to dissections, aortic valve disease, reoperations and mycotic aneurysms as well as teeth injuries were excluded. Five claims were excluded as the claims were not related to a negligence of AAA treatment.

Data from LÖF were extracted into a predefined protocol and further analyzed. The underlying diagnosis or symptoms, the management processing and the course of the suffering claimants was registered. LÖF covers all healthcare funded by the healthcare regions, which means that over 95% of all Swedish healthcare is covered by LÖF.

Injuries caused by negligence motivate economic compensation according to Patient's Injury Act (Patientskadelagen 1996;799). The sufferers' claims, available hospital records and the conclusions of the malpractice insurance review board concerning avoidable and compensated or not avoidable and thus not compensated, negligence claims were scrutinized by the authors.

The most serious negligence in each claim was listed. Further or secondary consequences of the complication were not included in this list. Other major injuries not related to the claimed complication were also recorded but not analyzed. The company's claims database was searched for ICD-10-SWE-codes related to AAA disease and for operation codes related to these diagnoses. All clinical data were extracted from the medical records of the claimants.

Background data on reported interventions during the actual 15-year period have been delivered from the yearly reports from the Swedish Vascular Registry (6) number of procedures, indication for interventions and complications.

The study has been approved by the Regional Ethics Committee, University of Uppsala (Acta 2018/015). No individual data are reported. It is not possible to trace individual patients, who have given a written consent when filing the claim with LÖF that the data could be used for research purposes.

## Results

From 2006 to 2020 there were 151 negligence claims related AAA. The material was separated in three 5-year cohorts: Period A 2006–2010 *n* = 43, Period B 2011–2015 *n* = 75, and Period C 2016–2020 *n* = 33. There were 85% males and 15% females. There was no difference in gender distribution between the treatment methods (females EVAR 19%, males 12% *p* = ns). The patients treated with EVAR were almost 3 years older than those treated with open surgery (71.7 ± 7.3 years versus 69.0 ± 7.1. *p* = 0.042).

In the SWEDVASC registry there were a total of 17,125 reported cases of AAA in 2006–2020 (Table 1). During this time there were registered 151 claims for negligence, 0.9% of all AAA in SWEDVASC. In the cohort subjected to EVAR (*n* = 8,507) 0.7% (*n* = 57) claimed a negligence and in the cohort subjected to open surgery (*n* = 8,618) 1.1% (*n* = 94) (*p* = 0.016) (Table 1).

**Table 1:** Number of procedures used in the three 5-year periods (A = 2006–2010, B = 2011–2015, C = 2016–2020) reported to SWEDVASC and number of filed negligence claims to the National Swedish Patient insurance company after surgery for aortic aneurysm surgery in 2006–2020. Claims Open versus EVAR: Period A versus B *p* = 0.0214, period B versus C *p* = ns.

Period	Total AAA	OPEN SWEDVASC *n*/%	OPEN claims *n*	OPEN claims %	EVAR SWEDVASC *n*/%	EVAR claims *n*	EVAR claims %
	SWEDVASC						
A	6,046	3,658/61	34	0.9	2,388/39	9	0.4
B	6,350	2,899/46	44	1.5	3,451/54	31	0.9
C	4,729	2,061/43	16	0.8	2,668/56	17	0.6
Total	17,125	8,618/50	94	1.1	8,507/50	57	0.7

The number of EVAR increased significantly between the periods A and C (*p* = 0.01) and the relative number of claims related to endovascular procedures increased between period A and B from 21 to 48% (*p* = 0.01) (Table 2).

**Table 2:** Treatment used in the three 5-year periods in filed claims with the National Swedish Patient Insurance Company after surgery for aortic aneurysm surgery in 2006–2020 (A = 2006–2010, B = 2011–2015, C = 2016–2020). Period A versus B *p* = 0.0214, period B versus C *p* = ns.

Period	Open *n*	EVAR *n*	Total *n*
A	34	9	47
B	44	31	77
C	16	17	34
Total	94	57	151

In 54% (82/151) of the AAA, the patients were without clinical symptoms at time for diagnosis. Those aneurysms were mostly identified when the individuals with abdominal symptoms were subjected to CT or ultrasound investigations (*n* = 55) or were included in a screening program (*n* = 27). There was an increasing number of claims over time related to asymptomatic and screening detected aneurysms (period A 28% versus B 63% (*p* = 0.001), (period B 63% vs. C 70% *p* = NS) (Table 3).

**Table 3:** The reason leading to diagnosis of AAA for 151 patients who have filed claims to the National Swedish Patient Insurance Company after surgery for aortic aneurysm surgery in 2006–2020 (A = 2006–2010, B = 2011–2015, C = 2016–2020).

Period	Asymptomatic	Symptomatic	Screening	Rupture	Miscellaneous	Not stated	Total
A	7	6	5	15	2	8	43
B	34	9	13	8	3	8	75
C	14	5	9	3	0	2	33
Total	55	20	27	26	5	18	151

The majority (100/151, 66%) of the negligence claims were judged being *not avoidable* and consequently not compensated for. Thirty-four of 94 claims (36%) after open surgery and 17 of 57 (30%) claims after EVAR were judged as *avoidable* and consequently compensated (*p* = ns). There was an increasing number of claims for avoidable injuries over time (period A versus period B 19% vs. 36% *p* = 0.047), (period B versus period C *p* = ns) (Table 4).

**Table 4:** Judged avoidability in 151 patients who have filed claims with the National Swedish Patient Insurance Company after surgery for AAA surgery in 2006–2020. Period A versus B *p* = 0.047, B versus C *p* = ns, A versus C = 0.002.

Period	Not avoidable (*n*/%)	Avoidable (*n*/%)	Total
A (2006–2010)	35/81	8/19	43
B (2011–2015)	48/64	27/36	75
C (2016–2020)	17/52	16/48	33
Total	100/66	51/34	151

Of the 82 claimants without symptoms of the aneurysm (55 asymptomatic and 27 screening detected) 31 (38%) experienced *avoidable* treatment injuries (Table 5). Claims after treatment of ruptured AAA, 10 of 26 (38%) were considered *avoidable*.

**Table 5:** Avoidability because of method of detection of AAA in 151 patients who have filed claims with the National Swedish Patient Insurance Company.

Symptoms	Not avoidable	Avoidable	Total
Asymptomatic elective	36	19	55
Symptomatic elective	16	4	20
Rupture	16	10	26
Screening detected	15	12	27
Miscellaneous	3	2	5
Not stated	14	4	18
Total	100	51	151

Eight of nine claims burn injuries were judged to be *avoidable* and were compensated (Table 6). Claims concerning lower leg circulation related to thromboembolism had compartment syndrome and rhabdomyolysis and were in 75% of the cases (9/13) considered *not avoidable* and not substituted. Seven of 17 (41%) claims recorded as intestinal ischemia after open surgery were *avoidable* and compensated and six after EVAR were all judged not *avoidable.*

**Table 6:** Claimed injury in relation to avoidability in 151 patients who have filed claims with the National Swedish Patient Insurance Company after surgery for AAA in 2006–2020.

Injury	Open			EVAR		
	Avoidable	Not avoidable	Total	Avoidable	Not avoidable	Total
Bleeding	1	2	3	2	0	2
Burn injury	6	0	6	2	1	3
Decubitus	6	3	9	0	0	0
Delayed diagnosis	0	1	1	1	2	3
Delayed treatment	1	1	2	0	1	1
Extremity ischemia	1	6	7	3	3	6
Intestinal ischemia	7	10	17	0	6	6
Graft infection	0	0	0	0	2	2
Miscellaneous	3	3	6	1	3	4
MOF	1	3	4	0	1	1
Motor nerve lesion	0	0	0	0	1	1
Sensory nerve lesion	1	3	4	0	0	0
Mental disorder	0	0	0	1	1	2
Renal artery occlusion	1	2	3	2	5	7
Renal insufficiency	0	0	0	1	1	2
Sexual dysfunction	0	6	6	0	0	0
Spinal cord ischemia	2	19	21	2	7	9
Stent problem	0	0	0	2	2	4
Stroke	1	0	1	2	2	4
Ureter lesion	4	0	4	0	0	0
Total	34	60	94	17	40	57

Spinal cord ischemia of varying magnitude from complete paraparesis to peroneal paresis was considered as *not avoidable* in 26 of 30 claims (87%). The spinal lesions had additive claims as abdominal compartment syndrome (*n* = 3) and extremity compartment syndrome (*n* = 6). Urinary incontinence and sexual dysfunction were secondary consequences of spinal cord ischemia in five patients. Sexual dysfunction without signs of spinal cord ischemia was claimed in six patients, all after open surgery and regarded as *not avoidable*. Stroke was in one patient initiated by a too early anticoagulant treatment. Multiorgan failure (MOF) occurred in five patients were all but one due to *not avoidable* negligence.

## Discussion

This analysis is based on the claimants-sufferers' experience during the care for an abdominal aortic aneurysm, and it gives information on the motivation – background for caretakers to sign a claim of negligence and request economic compensation for their suffering and financial losses, and it is not an information on the magnitude of complications after invasive treatment of aortic aneurysms.

The number of negligence claims to the Swedish Insurance Board was less than one per cent of all AAA treatments during this 15-year period.

During the 15-year period covered in this analysis the number of invasive treatments for aortic aneurysm registered in SWEDVASC has decreased (from 6,046 in period A to 4,729 period C) (27%). The proportion treated endovascularly has increased from 2,388 to 2,668 (from 39 to 56% period A to period C). This is in conformity with recent international communications (1).

The overall negligence claim rate has been fairly stable. Relatively seen less claims have been raised from patients treated with endovascular procedures at 0.7% and versus open 1.1% Several studies have compared open and endovascular treatment of AAA. A recent meta-analysis has shown endovascular treatment to be superior in the short time perspective, but late complications were more frequent and long-term survival being similar (1, 7).

From the patients’ side there is no restriction for when a claim can be raised. The patient can be economically compensated whether there is negligence or maltreatment, the system for financial compensation being separated from the medicolegal system. This is an obvious benefit for the sufferer. In almost one third ((36%) 51 of 151) of the negligence claims, the claimed injury was judged avoidable, and the sufferer therefore received economic compensation.

According to Swedish law an avoidable injury can occur despite of care being given in accordance with good professional norms, modern medical standards and scientific evidence. Such an injury motivates economic compensation for the sufferer and the economic inconvenience the claimant has reported. Many complications after invasive treatment of AAA, both open and endovascular are considered as calculated risk for all patients, and the patients have to be well informed both regarding these risks and when they can expect economic compensation for suffering and economic loss. The responsible surgeon in charge must inform the sufferers of their legal rights.

In this study, the monetary amount given to the patients is not reported as this also includes individual and socioeconomic considerations, and this also makes it less meaningful to make comparisons between countries with different rules and reimbursement systems. The main point is that patients have the legal right to receive economic compensation if they experience a lesion or complication, which is considered avoidable by a review board of experts.

At present, there are no data on how many avoidable complications that are not being claimed. This would need an analysis of patient journals of all interventions. Performing such a study prospectively with a well-defined protocol would on the other hand require inclusion of all interventions for several years ahead and is hardly practical or feasible.

When analyzing the list of the negligence claims separately, spinal cord ischemia dominates, after both open surgery and after EVAR. Only four were avoidable and therefore compensated. The claimant experiencing this tragic consequence (in a worst case scenario paraparesis) without compensation certainly leads to frustration. Routine spinal cord drainage is a way to diminish this risk (1, 8).

Next in frequency is intestinal ischemia resulting in abdominal compartment syndrome and renal failure. Seven were judged avoidable and compensated after open surgery. Third in frequency was extremity ischemia with rhabdomyolysis and fasciotomy and four were judged avoidable.

Of special interest is individuals with non-symptomatic AAA, who after intervention, open or endovascular, come out with a complication of great clinical significance as paraparesis due to spinal cord ischemia but did not get economic compensation as the lesion was judged non-avoidable. This is in contrast to claimants with a slightly bad-looking scar after an avoidable burn injury during surgery for a life-threatening ruptured AAA, which motivated economic compensation.

One potential problem with the background data from the Swedish vascular registry is changes in the protocol between the two last 5-year periods. However, this does not influence the total number of invasive treatments, and the coverage of the registry is more than 95% (3). Moreover, this change should not have any influence on negligence claims. The frequency of claims in this study on abdominal aortic aneurysm is rather similar to that in a previous study analyzing all types of arterial interventions during a shorter time span (5).

Performing invasive treatment will never go without complications. Hopefully, the number of avoidable injuries will decrease over time. It is important that the profession adheres to current guidelines (1), and continuously analyses adverse events in morbidity and mortality conferences, a crucial motivation being to learn from mistakes, the ultimate goal being to make patient care safer. It is also important that senior surgeons take their responsibility in education and in how to avoid problems as far as possible. The frequency of negligence claims judged as not avoidable might reflect the experience of the vascular team performing the intervention, and the increasing centralization to larger units, which have a continuous impact (1).

In conclusion, between 2006 and 2020 there were more than 17,000 interventions for AAA in Sweden, and in this population a negligence after open surgery was claimed in 1.1% and in 0.7% after EVAR. One third of these negligence claims were judged as avoidable and the claimants were economically compensated for their suffering. The negligence claims judged as non-avoidable are calculated risks after invasive intervention, and the patient must be well informed on the risks after this type of treatment. Avoiding complications and decreasing risks are continuous challenges for the health care sector.
